# Supplementary material for: Carbon Nanotube-Based Electrochemical Biosensor for Label-Free Protein Detection
Source: Biosensors (Basel). 2019 Dec 17;9(4):144. doi: 10.3390/bios9040144 (PMC6956173; doi:10.3390/bios9040144)
Supplement: Supplementary file 1 [file biosensors-09-00144-s001.pdf]

Table S1. Source code for programmed Arduino Uno ohmmeter unit.

```
int analogPin = 0;
int raw = 0;
int Vin = 5;
float Vout = 0;
float knownR = 1000;
float sensorR = 0;
float buffer = 0;
float R1 = 0
float R2 = 0
float R3 = 0
float R4 = 0
float R5 = 0

void setup()
{
Serial.begin(9600);
}

void loop()
{
raw= analogRead(analogPin);
if(raw)
{
buffer = raw * Vin;
Vout = (buffer)/1024.0;
buffer = (Vin/Vout) -1;
sensorR = knownR * buffer;
if (sensorR == R1) {
if (R2 == sensorR) {
R3 = sensorR;
} else{
R2 = sensorR;
}
} else {
R1 = sensorR;
}
Serial.print("Resistance: ");
Serial.println(sensorR);
if ((R1 != 0) && (R1 == R2 == R3)) {
    delay(100000)
} else {
delay(2000);
}}}
```
